# Faecal Carriage of Multidrug Resistant *Enterobacterales* and Associated Factors among Neonates Admitted at Tertiary Hospital in Dar es Salaam, Tanzania

**DOI:** 10.24248/eahrj.v8i3.804

**Published:** 2025-01-30

**Authors:** Hadija A. Salega, Doreen Kamori, Upendo O. Kibwana, Joel Manyahi, Agricola Joachim, Salim Masoud, Ambele M. Mwandigha, Mariam Mirambo, Martha F. Mushi, Stephen E. Mshana, Mtebe V. Majigo

**Affiliations:** a Department of Microbiology and Immunology, Muhimbili University of Health and Allied Sciences, Dar es Salaam, Tanzania; b Department of Microbiology and Immunology, Catholic University of Health Allied Sciences, Mwanza, Tanzania.

## Abstract

**Background:**

Hospitalised neonates are at increased risk of carrying extended-spectrum β-lactamase-producing *Enterobacterales* (ESBL-PE) and carbapenemase-producing *Enterobacterales* (CPE), possibly leading to invasive infections. This study determined the faecal carriage of ESBL-PE, CPE, and associated factors among neonates at Muhimbili National Hospital (MNH).

**Methods:**

A hospital-based cross-sectional study was conducted among neonates aged ≤ 28 days admitted at MNH. The participants’ data and rectal swab samples were collected. Samples were processed to detect ESBL-PE and CPE. Results were confirmed using the double-disc diffusion synergy test and modified carbapenem inactivation method, respectively. An antimicrobial susceptibility test was performed using the Kirby Bauer disk diffusion method.

**Results:**

Three hundred forty neonates with a median age of 3 days (IQR: 2–9) were enrolled. The carriage rate of ESBL-E and CPE was 39.4%(134/340) and 1.8%(6/340), respectively. Klebsiella pneumoniae (66.9%) and Escherichia coli (66.7%) were the common isolates for ESBL-PE and CPE, respectively. The factors independently associated with ESBL-PE carriage were antibiotic use (aOR 2.73, 95% CI: 1.38–5.39, *p=.04*), age increase (aOR 1.09, 95% CI: 1.02–1.15, *p=.006*), prolonged hospitalisation (aOR 2.92, 95% CI: 1.17–7.29, *p=.02*), and neonate-sucking their fingers (aOR 2.98, 95% CI: 1.04–8.58, *p=.04*). The study observed a trend of CPE carriage toward neonates with prolonged hospitalisation (*p=.05*). ESBL-PE low resistance was observed to meropenem (0.9%), amikacin (2.7%-6.7%), and gentamicin (19.4% to 100 %).

**Conclusions:**

The study revealed a relatively high carriage rate of multidrug resistant Enterobacterales among neonates admitted to a tertiary hospital. These findings underscore the importance of continuous surveillance of ESBL-PE and CPE to prevent infections and limit their potential transmission within hospital settings and the community.

## BACKGROUND

The extended-spectrum beta-lactamase-producing *Enterobacterales* (ESBL-PE) and carbapenemase-producing *Enterobacterales* (CPE) are significant global public health concerns. The World Health Organization (WHO) has classified ESBL-PE and CPE as critical priority pathogens for research.^1^ Carrying ESBL-PE and CPE is a threat because the encoding genes on plasmids rapidly spread in the bacterial population and act as a potential reservoir for acquiring ESBL-PE and CPE.^2–4^ In addition, ESBL-PE and CPE can readily colonise the gastrointestinal tract of the new born and may lead to neonatal infections.^5^

Globally, the carriage rate of ESBL-PE among healthy individuals in 2015 was 14%, with an increased annual rate of approximately 5%.^6^ Africa has one of the highest ESBL-PE carriage rate, and is attributed to factors such as; high population density, limited access to clean drinking water, and widespread poverty.^7^ For example, infants and neonates in Morocco had a 58% faecal carriage rate of ESBL-PE.^8^ In Tanzania, two studies reported ESBL-PE carriage rates of 54.6% and 25.4% among neonates.^5,9^ In addition, a study among children found ESBL-PE carriage rate of 11.6% and 50.4% in the community and hospital settings, respectively.^10^ Significant carriage of CPE has also been reported in Africa. For example, a study in Angola reported a CPE prevalence of 27.4 % among hospitalised children,^11^ while in Morocco, a study among neonates reported a carriage rate of 1.8%.^8^ In addition, a study in Algeria showed a CPE carriage rate of 1.6%.^12^ Previous studies have reported several factors associated with the carriage of ESBL-PE and CPE, including hygiene, prolonged hospital stays, low birth weight, invasive procedures, underlying diseases, and antibiotics use.^5,12–15^

These observations indicate that ESBL-PE and CPE carriage among neonates is a continuous threat, and the magnitude differs from one setting to another. This study determined the faecal carriage rate of ESBL-PE and CPE and associated factors among neonates admitted at Muhimbili National Hospital (MNH), a tertiary hospital in Dar es Salaam, Tanzania.

## METHODS

### Study Design and Setting

A hospital-based cross-sectional study was conducted from February to May 2020 at MNH, the largest tertiary hospital in Tanzania. MNH serves as a university teaching hospital with 1,500 bed capacity and attends to approximately 2,000 outpatients daily. MNH have more than 100 bed capacities for neonatal intensive care unit (NICU) and neonatal wards. On average, 15 to 20 neonates are admitted daily, translating to approximately 450 to 500 admissions per month.

### Study Population, Sample Size, and Sampling Procedure

The study enrolled neonates aged ≤ 28 days whose parents/guardians consented to participate, excluding neonates with severe congenital malformations such as anal atresia. The sample size was calculated using the Kish Leslie formula for cross-sectional studies, considering a 25.4% prevalence of ESBL-PE reported in a study in Tanzania^9^ and 27.4% for CPE reported in Luanda, Angola.^11^ The minimum sample size required was 291 for ESBL-PE prevalence, and 306 for CPE prevalence. A total of 340 neonates were conveniently recruited from the NICU and neonatal wards to meet the study objectives.

### Data Collection

A structured data collection tool was used to collect socio-demographic information, hygienic behavioural characteristics, and clinical information of paired neonates and their mothers. Socio-demographic information included; age, sex, physical address, parents’ or guardians’ level of education, and neonates’ birth weight. The mother’s hygienic behavioural characteristics included; toilet use, food handling, hand washing, and new born bathing practices. Clinical information of neonates included; history of antibiotic use, mode of delivery, invasive procedures, duration of hospital exposure, previous hospitalisation, gestation age, and comorbidities. Maternal clinical information collected encompassed sexually transmitted infections (STI) such as; Human Immunodeficiency Virus (HIV) and Syphilis, other comorbidities (e.g. pre-eclampsia, eclampsia, anaemia, gestational diabetes), and history of antibiotic use during pregnancy.

### Study Variables

Birth weight below 2.5 kg was classified as low birth weight. Pre-term birth was defined as a gestational age at birth of less than 37 weeks. Hospital stay referred to the duration neonates spent in hospital beds during the study period. Previous hospitalisation was defined as any hospital admission occurring within a specified time prior to neonate’s current hospitalisation. Sexually transmitted infections (STI) and other comorbidities like (preeclampsia, eclampsia, anaemia, and gestational diabetes) during pregnancy may contribute to the presence of antimicrobial resistance organisms. Pregnant women with these conditions often require close monitoring from healthcare workers, hence frequent hospital visits or hospitalisation, hence increasing their exposure to contaminated hospital environments or items, potentially transmitting resistance genes to their new-borns. Multi-drug resistance (MDR) bacteria is defined as antimicrobial resistance expressed by a bacteria to at least one antimicrobial drug in three or more antimicrobial classes. ESBL-PE are Gram-negative bacteria in the human gastrointestinal tract. They can produce ESBL enzymes that confer resistance to different beta-lactam antibiotics, including first, second, third, and fourth-generation cephalosporins, as well as aztreonam. CPE are Gram-negative bacteria that produce enzymes (*carbapenemase*) that inactivate carbapenems (meropenem, imipenem, doripenem, and ertapenem), and several other classes of beta-lactam antibiotics such as penicillin, cephalosporin, and monobactam.

### Sample Collection and Laboratory Procedures

The rectal swab was collected by gently inserting a moist sterile swab approximately 1 to 2 inches into the anal canal. The swab was then inserted into Cary-Blair transport media and transported to the microbiology laboratory at MUHAS to process and identify the bacteria.^16^ For screening ESBL-PE and CRE, the rectal swabs were inoculated onto two MacConkey agar (MCA) (Oxoid Ltd UK); one supplemented with ceftazidime at 2 μg/ml and another supplemented with meropenem at 1 μg/ml for ESBL-PE and CRE screening, respectively. Then, plates were incubated aerobically at 37 ºC for 18 to 24 hours.^17,18^

Isolates were subcultured on Nutrient Agar (NA) for further analysis, including conventional biochemical tests such as oxidase, urease, citrate utilisation, Kligler Iron Agar (KIA), and SIM tests for bacterial identification.^19^ In addition, Analytical Profile Index (API-20E, bioMérieux, Marcy-l’Étoile, France) test was used to identify inconclusive isolates. Finally, isolates were confirmed as ESBL-PE using the Double-Disc Diffusion Synergy Test (DDST) and as CPE using the Modified Carbapenem Inactivation Method (mCIM).^20,21^ In addition, Antimicrobial Susceptibility Testing (AST) was performed using the Kirby Bauer disk diffusion method.^22^ Antimicrobial disks, meropenem (10µg), amikacin (30µg), gentamicin (10 μg), amoxicillin/clavulanic acid (20/10 μg) and ciprofloxacin (5 µg) were used for ESBL-PE isolates. Regarding antimicrobial susceptibility pattern for CPE aztreonam (30 µg), ciprofloxacin (5 µg), amikacin (30µg), gentamicin (10 μg), cefepime 30 µg and amoxicillin/clavulanic acid (20/10 μg) antimicrobial disks were used for testing CPE isolates.^23^ The inhibition zones were measured and interpreted according to the Clinical Laboratory Standard Institute (CLSI) 2017 guideline.

### Quality Control

The culture media were prepared following the manufacturer’s guidelines and subjected to quality-control to ensure performance and sterility. A 0.5 McFarland standard was used to standardise the turbidity of bacterial suspensions for ESBL-PE and CPE testing. As per CLSI guidelines, *Klebsiella pneumoniae* (ATCC-700603) and *E.coli* (ATCC-25922) were used as positive and negative control bacteria strains, respectively, for ESBL-PE. Also, for CPE, *K.pneumoniae* (ATCC-1705) and *E.coli* (ATCC-25922) were used as positive and negative control bacteria strains, respectively. ^21^

### Data Analysis

Descriptive analysis was performed using medians with interquartile range (IQR) for continuous variables and proportions for categorical variables. Univariable and multivariable logistic regression models were used to determine the factors independently associated with faecal carriage of ESBL-PE. Factors with a *p*-value of less than 0.1 were subjected to multivariable logistic regression analysis using a backward stepwise elimination method noting their Odds Ratios (OR) and 95% Confidence Intervals (CI). Fisher’s exact test was used to determine the factors associated with faecal carriage of CPE. A *p*-value of ≤.05 was considered statistically significant for all the statistical tests.

### Ethical Consideration

The study obtained ethical approval from the Institutional Review Board (IRB) of the Muhimbili University of Health and Allied Sciences (MUHAS) with reference number DA.287/298/01A. Written informed consent was obtained from the mothers or guardians of each study participant. For neonates identified with drug-resistant strains, the findings were promptly shared with the attending clinicians for proper action.

## RESULTS

### Participant’s Socio-Demographic, Hygienic, and Clinical Characteristics

A total of 340 neonates were enrolled, with a median age of 3 days (interquartile range (IQR) 2–9) and median birth weight of 2.01 kg (IQR: 1.43–2.84). The majority, 185/340 (54.4%), were males, 243/340 (71.5%), were aged ≤ 7 days, and 220/340 (64.7%) had a birth weight of < 2.5 kg. About 98 % of the participants were delivered at the hospital, and 62.7% were admitted to the NICU (Table 1).

**Table 1:** Socio-Demographic, Hygiene, and Clinical Characteristics of Study Participants (N=340)

Variable	Number	Percent %
Age in days		
Median 3 (IQR: 2–9) days		
≤ 7 days	243	71.5
> 7 days	97	28.5
Sex		
Female	155	45.6
Male	185	54.4
Birth weight		
Median 2.01 (IQR:1.43–2.84) Kg		
< 2.5 Kg	220	64.7
≥ 2.5 Kg	120	35.3
Place of birth		
Hospital	334	98.2
Home	6	1.8
Mother's/guardian's level of education		
No formal education	5	1.4
Primary	143	42.1
Secondary	169	49.7
College/University	23	6.8
Ward		
NICU	213	62.7
Other neonatal wards	127	37.3
Residence		
Kigamboni	25	7.4
Ubungo	36	10.6
Temeke	113	33.2
Kinondoni	41	12.1
Ilala	99	29.1
Others	26	7.6
Hygienic practices		
Shared community toilet	96	28.2
Shared family toilet	220	64.7
Self-contain toilet	24	7.1
Bathing practices of the newborn		
Source of water	24	7.1
Stagnant water source	23	95.8
Running water	1	4.2
Use medicated soap	13	54.2
Neonate had contact with other people		
Yes	20	5.9
No	320	94.1
Neonate sucking fingers		
Yes	25	7.3
No	315	92.7
Use of antibiotic		
Yes	235	69.1
No	105	30.9
Previous history of hospitalization		
Yes	100	29.4
No	240	70.6
Duration of current hospitalization		
≤ 7 days	287	84.4
> 7 days	53	15.6
Gestation age at birth		
Pre-term	230	67.7
Full term	110	32.3
Mode of delivery		
Spontaneous vaginal delivery	189	55.6
Assisted delivery	5	1.5
Caesarean section	146	42.9
Mother's antibiotic use		
Yes	108	31.8
No	232	68.2
Mother HIV status		
Positive	19	5.6
Negative	321	94.4

IQR-interquartile range, Kg-Kilogram, NICU-Neonatal intensive care unit

The majority of the neonates’ mothers, 220/340 (64.7%), shared toilets with family members, while 96/340 (28.2%) shared with the community, and 24/340 (7.1%) had self-contained toilets. Twenty-four neonates (7.1%) were bathed, and of these, 23/24 (95.8%) used stagnant water, and 13/24 (54.2%) used medicated soap. A total of 320/340 (94.1%) neonates did not have physical contact with other people besides their mothers and hospital caregivers. Moreover, 25/340 (7.3%) neonates were sucking fingers (Table 1).

### Neonates and Mothers’ Clinical Information

The study observed that 235 (69.1%) neonates had used antibiotics such as ampiclox, gentamicin, and ceftriaxone either once or more than once during their hospital stay. Furthermore, 100/340 (29.4%) neonates had a history of previous hospitalisation before the current admission. The majority, 287/340 (84.4%) neonates, were hospitalised for ≤ 7 days. In addition, a total of 230/340 (67.7%) neonates had a premature birth, and 189/340 (55.6%) were delivered by Spontaneous Vaginal Delivery (SVD) (Table 1).

We observed that 232/340 (68.2%) mothers did not use antibiotics during pregnancy, and regarding HIV status, the majority, 321/340 (94.4%), were negative for HIV (Table 1). In addition, mothers of the study participants were diagnosed with several comorbidities during the current pregnancy, with UTI being the most frequent comorbidity, accounting for 26.5%, followed by preeclampsia at 20%, malaria at 4.4%, eclampsia at 4.1%, and chronic hypertension 2.9.

### Faecal Carriage of ESBL-PE and CPE

**Figure 1:**
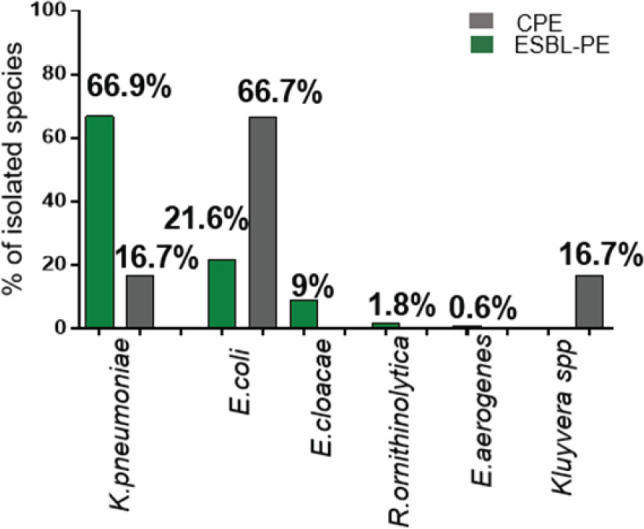
Faecal Carriage of ESBL-PE and CPE among Neonates

Faecal carriage of ESBL-PE was detected in 134/340 (39.4%) neonates, whereby 166 *Enterobacterales* were isolated. The most commonly isolated ESBL-PE was *K. pneumoniae* 111/166 (66.9%), followed by *E. coli*, 36/166 (21.7%). Faecal carriage of CPE was detected in 6/340 (1.8%) neonates, whereby three different species were isolated. The most commonly isolated CPE was *E. coli* 4/6 (66.7%), followed by *K. pneumoniae* 1/6 (16.7%) and *Kluyvera* spp 1/6 (16.7%) (Figure 1).

It illustrates the percentage distribution of the isolated ESBL-PE and CPE bacteria species

### Factors Associated with Faecal Carriage of ESBL-PE and CPE

On univariable logistic regression analysis, it was observed that when age increased by one day, the odds of carrying ESBL-PE increased by 1.17 times (cOR 1.17, 95% CI: 1.12–1.22, *p<.001*). The neonates admitted to the NICU had 1.54 odds of carrying ESBL-PE than those admitted to other neonatal wards (Table 2). Giving a bath to a newborn had five times the odds of carrying ESBL-PE (cOR 5.17, 95% CI: 1.99–13.40, *p=.001*) compared to neonates who did not receive a bath. A neonate-sucking finger was approximately three times more likely to carry ESBLPE than neonates who did not suck their fingers (cOR 2.97, 95% CI: 1.27–6.93, *p=.012*) (Table 2). The neonates who used antibiotics were five times more likely to have ESBL-PE (cOR 5.13, 95% CI: 2.88–9.16, *p<.001*) than those who did not. Furthermore, neonates with a history of hospitalisation were approximately three times more likely to carry ESBL-PE (cOR 2.81, 95% CI 1.74–4.53, *p<.001*). Neonates hospitalised for > 7 days were eight times more likely to carry ESBL-PE (cOR 8.09, 95% CI: 3.98–16.44, *p<.001*) compared to being hospitalised for ≤ 7 days. Premature neonates had 1.72 times more likely to carry ESBL-PE than full-term neonates (cOR 1.72, 95% CI: 1.06–2.78, *p=.027*). In addition, neonates whose mothers were HIV positive were four times more likely to carry ESBL-PE than mothers who were HIV negative (cOR 4.69, 95% CI: 1.65–13.35, *p=.004*) (Table 2).

**Table 2:** The Socio-Demographic, Hygiene, and Clinical Characteristics Associated with Fecal Carriage of ESBL-PE among Neonates

Variable	Fecal carriage of ESBL-PE n (%)	cOR (95%CI)	*p-value*	aOR (95%CI)	*p-value*
Age					
Age in days	134 (39.4)	1.17 (1.12–1.22)	<.001	1.09 (1.02–1.15)	.006
Ward					
NICU	92 (43.2)	1.54 (0.97–2.43)	.065	2.16 (0.90–5.21)	.09
Other neonatal wards	42 (33.1)	ref		ref	
Bath the newborn					
Yes	18 (75.0)	5.17 (2.00–13.40)	.001	1.12 (0.35–3.66)	.85
No	116 (36.7)	ref		ref	
Neonate sucking fingers					
Yes	16 (64.0)	2.97 (1.27–6.93)	.012	2.98 (1.04–8.58)	.04
No	118 (37.5)	ref		ref	
Use of antibiotic					
Yes	117 (49.8)	5.13 (2.88–9.16)	<.001	2.73 (1.38–5.39)	.04
No	17 (16.2)	ref		ref	
Previous history of hospitalisation					
Yes	57 (57.0)	2.81 (1.74–4.53)	<.001	1.30 (0.68–2.47)	.43
No	77 (32.1)	ref		ref	
Duration of current hospitalisation					
>7 days	42 (79.3)	8.09 (3.98–16.44)	<.001	2.92 (1.17–7.29)	.02
≤7 days	92 (32.1)	ref		ref	
Gestation age at birth (weeks)					
Preterm	100 (43.5)	1.72 (1.06–2.78)	.027	1.20 (0.48–2.99)	.70
Full term	34 (30.9)	ref		ref	
HIV status					
Positive	14 (73.7)	4.69 (1.65–13.35)	.004	2.48 (0.67–9.11)	.17
Negative	120 (37.4)	ref		ref	

ESBL-PE= extended spectrum beta-lactamase producing Enterobacteralaes, cOR = crude Odds Ratio, aOR = adjusted Odds Ratio, NICU = Neonatal Intensive care unit, HIV=human immunodeficiency virus

On multivariable logistic regression analysis, it was observed that age (aOR 1.09, 95% CI: 1.02–1.15, *p=.006*) was independently associated with faecal carriage of ESBL-PE. The use of antibiotics (aOR 2.73, 95% CI: 1.38–5.39, *p=.04*), hospitalisation for >7 days (aOR 2.92, 95% CI: 1.17–7.29, p=.02), and neonate sucking their fingers (aOR 2.98, 95% CI: 1.04–8.58, *p=.04*) were also independently associated with faecal carriage of ESBLPE (Table 2). In this study, only prolonged hospitalisation showed a trend toward association with CPE carriage (*p=.05*)

### Antibiotic Resistance Pattern of ESBL-PE and CPE

ESBL-PE showed variable antibiotic resistance to gentamicin (19.4%-100 %) and amoxicillin/clavulanic acid (11.1%–100%). The least antibiotic resistance was observed in meropenem, whereby only 1(0.9%) *K. pneumoniae* isolate was resistant. Resistance to amikacin was low (2.7%-6.7%). Resistance to ciprofloxacin was observed among *E. aerogenes* 1(100%), *R. ornithinolytica* (66.7%), *K. pneumoniae* 28(25.2%), *E. coli* 14(38.9%) and *E. cloacae* 4(26.7%). Among ESBL-PE isolates, *K. pneumoniae* was found to resist most antibiotics tested, while *R. ornithinolytica* had the least resistance compared to all other isolates (Table 3).

**Table 3:** Antimicrobial Resistance Pattern Among ESBL-PE and CPE isolates

ESBL-PE isolates	Meropenem	Ciprofloxacin	Gentamicin	Amoxacilin/Clavulanic acid	Amikacin	Cefepime	Aztreonam
	n (%)	n (%)	n (%)	n (%)	n (%)	n (%)	n (%)
K. pneumoniae (n=111)	1 (0.9)	28 (25.2)	73 (65.8)	35 (31.5)	3 (2.7)	NA	NA
E. coli (n=36)	0	14 (38.9)	7 (19.4)	4 (11.1)	0	NA	NA
R. ornithinolytica (n=3)	0	2 (66.7)	1 (33.3)	0	0	NA	NA
E. cloacae (n=15)	0	4 (26.7)	10 (66.7)	14 (93.3)	1 (6.7)	NA	NA
E. aerogenes (n=1)	0	1 (100)	1 (100)	1 (100)	0	NA	NA
CPE isolates							
K. pneumoniae (n=1)	NA	1 (100.0)	0	1 (100.0)	0	1 (100.0)	1 (100.0)
E. coli (n=4)	NA	4 (100.0)	4 (100.0)	4 (100.0)	0	4 (100.0)	4 (100.0)
Kluyvera spp (n=1)	NA	1 (100.0)	0	1 (100.0)	0	1 (100.0)	1 (100.0)

NA indicates not applicable

All CPE isolates showed (100%) resistance to tested drugs such as ciprofloxacin, cefepime, amoxicillin/clavulanic acid, and aztreonam. Furthermore, *E. coli* showed 100% resistance to gentamicin. However, all isolates were sensitive to amikacin (Table 3).

## DISCUSSION

The present study has revealed that ESBL-PE faecal carriage is 39.4% in neonates admitted to tertiary hospitals in Tanzania. These findings concur with findings obtained in a study conducted at Arba Minch General Hospital in Ethiopia, which reported the carriage of ESBL of 34%.^24^ The current study demonstrated a relatively low faecal carriage of ESBL-PE compared to the 56% reported previously in Dar es Salaam, Tanzania, and in another study done in Cape Town, South Africa, at Red Cross War Memorial Children’s Hospital that reported a higher faecal carriage of ESBL-PE of 48%.^25,26^ The possible reason for the variation between this study and other studies may be due to differences in the clinical conditions of the study participants and geographical variation. For example, a study done in Tanzania included children hospitalised because of fever. Geographical variation may also account for the difference in results between this study and a study done in South Africa.^25,26^

Our study has demonstrated that the most common isolated ESBL-PE bacteria was *K. pneumoniae,* similar to previous studies in Morocco, Kenya, Mwanza – Tanzania, South Africa, and Ghana.^5,8,26–28^ *E. coli* was the second most isolated bacteria in this study, in contrast to observation in a previous study done in Saudi Arabia, which found that the predominant bacteria isolated was *E. coli.* ^29^ These variations may be due to the difference in the geographical area. Other isolated bacteria in this study were *E. cloacae* and *E. aerogenes;* these findings are similar to findings of a similar study.^5^ The current study also reports the isolation of *R. ornithinolytica*. This is an important organism to note as it is rarely isolated in humans, and it is found to be hospital-acquired and frequently occurs in immunocompromised patients.^30,31^

Our analysis of the factors associated with ESBL-PE carriage revealed that antibiotic use is associated with ESBL-PE carriage; this is similar to findings reported in previous studies.^5,14,32^ This association is possible since many admitted neonates were empirically treated with antibiotics either once or multiple times during their hospital stay. Such treatments can disrupt the establishment of normal intestinal bacterial flora (gut microbiota) and expose bacteria to the drugs.^5^ Furthermore, this exposure may initiate the development of resistance mechanisms such as ESBL enzymes and increase the risk of colonisation with ESBL-PE.

Neonates of older age are independently associated with faecal carriage of ESBL-PE in the present study; this is comparable to findings in the previous studies.^27^ The possible explanation is that as age increases, neonates become more likely to be exposed to the hospital environment, have frequent antibiotic use, increased contact with healthcare workers, and invasive procedures that make them more likely to carry the multi-drug resistant organisms. Furthermore, this study reports the association between ESBL-PE carriage and prolonged hospital stay, similar to previous studies in Ghana and Malaysia.^14,33^ This is because the longer duration of neonates’ stay in the wards exposes them to a higher risk of carrying ESBL through exposure to various objects/items that may be contaminated.

The study reports the association between ESBL-PE carriage and neonate-sucking their fingers; this is comparable to a previous study conducted in India.^34^ However, this factor is rarely documented as a risk factor for the carriage of ESBL-PE. A plausible explanation for this association is that neonates’ fingers may come into contact with ESBL-PE contaminated environments, objects, or healthcare workers within the hospital setting, facilitating the transmission of resistant bacteria.

The present study also observed low resistance to meropenem and amikacin for ESBL-PE isolates. This finding is similar to results reported by previous studies conducted in Lebanon, Tanzania, and Indonesia.^5,17,35^ The possible explanation is that meropenem and amikacin are not frequently prescribed antibiotics in our local setting, as meropenem is a reserved drug for confirmed multi-drug resistant bacterial infections. On the other hand, high resistance to ciprofloxacin, gentamicin, and amoxicillin-clavulanic acid against ESBL-PE was observed. This finding is similar to the reports of previous studies conducted in Mwanza, Tanzania, and Addis Ababa, Ethiopia.^5,36^

This study has demonstrated a CPE faecal carriage of 1.8% among admitted neonates. This finding is similar to findings of previous studies done in Morocco, Algeria, and Ethiopia, which found faecal carriage of 1.8%, 1.6%, and 2.4%, respectively.^8,12,24^ In contrast, other studies have reported higher faecal carriage of CPE, ranging from 7.9 to 27.4%.^11,14,37^ The possible explanation for these variations could be; different CPE detection techniques, the type of specimens used, and the study population’s characteristics (age and exposure). For example, studies conducted in Luanda and Morocco used ChromoID media and molecular techniques to screen and confirm CPE.^11,37^ Furthermore, this study’s carriage is lower than that reported in previous studies among hospitalised children and adults.^11,37^ This is possible because adults are more likely to have been exposed to risk factors for carriage of CPE, including previous hospitalisation, prolonged hospitalisation, and antibiotic use, including self-prescription of antibiotics.

In this study, three different CPE species were isolated, with *E. coli* being the predominant species. This finding is similar to the previous study done in Luanda, which found that *E. coli* was the most common isolated species.^11^ Other species were *K. pneumoniae* and *Kluyvera* spp. This observation concurs with findings from previous studies.^12,38^ The bacterium *Kluyvera* spp is associated with urinary tract infections and sepsis with multi-organ failure in hospitalised patients.^39^

We report that prolonged hospitalisation shows a trend toward the association with the carriage of CPE among neonates. This finding is similar to the study done in Ghana, which found that the carriage rate increased with the duration of hospitalisation from 13% for neonates screened on day 1 of admission to 42% by day 2, 47% by day three, and reached a plateau at 91% by day 15.^14^ This is likely because neonates with prolonged hospitalisation are more exposed to the hospital environment and invasive procedures.

Our study has demonstrated a high resistance of CPE isolates to commonly used antibiotics such as ciprofloxacin, aztreonam, amoxicillin-clavulanic acid, and cefepime and moderate resistance to gentamicin. These findings concur with previous studies,^40,41^ however, all isolates were sensitive to amikacin. This finding is similar to that reported in earlier studies done in Shanghai and Algeria, where most isolates were susceptible to amikacin.^40,41^

## CONCLUSIONS AND RECOMMENDATIONS

This study revealed a relatively high faecal carriage of ESBL-PE and CPE among neonates suggesting an increased risk of resistant bacteria transmission in hospital settings. In addition, most isolates were resistant to commonly prescribed antimicrobial agents. These findings underscore the urgent need to strengthen infection prevention and control measures and enhance antimicrobial stewardship programs. Continuous surveillance of ESBL-PE and CPE among neonates is also crucial to prevent infections and potential transmission to other patients and healthcare workers within hospital settings.

### Study Limitation

It was not possible to perform the genotypic tests for molecular characterisation of ESBL-PE and CPE genes due to budget constraints. However, the isolates have been stored at −80° centigrade for further analysis once funds become available.
